# A genome-wide association study of the longitudinal course of executive functions

**DOI:** 10.1038/s41398-021-01510-8

**Published:** 2021-07-10

**Authors:** Bernadette Wendel, Sergi Papiol, Till F. M. Andlauer, Jörg Zimmermann, Jens Wiltfang, Carsten Spitzer, Fanny Senner, Eva C. Schulte, Max Schmauß, Sabrina K. Schaupp, Jonathan Repple, Eva Reininghaus, Jens Reimer, Daniela Reich-Erkelenz, Nils Opel, Igor Nenadić, Susanne Meinert, Carsten Konrad, Farahnaz Klöhn-Saghatolislam, Tilo Kircher, Janos L. Kalman, Georg Juckel, Andreas Jansen, Markus Jäger, Maria Heilbronner, Martin von Hagen, Katrin Gade, Christian Figge, Andreas J. Fallgatter, Detlef E. Dietrich, Udo Dannlowski, Ashley L. Comes, Monika Budde, Bernhard T. Baune, Volker Arolt, Ion-George Anghelescu, Heike Anderson-Schmidt, Kristina Adorjan, Peter Falkai, Thomas G. Schulze, Heike Bickeböller, Urs Heilbronner

**Affiliations:** 1 Department of Genetic Epidemiology, University Medical Center Göttingen, Georg-August-University Göttingen, Göttingen, 37073 Germany; 2 Institute of Psychiatric Phenomics and Genomics (IPPG), University Hospital, LMU Munich, Munich, 80336 Germany; 3 Department of Psychiatry and Psychotherapy, University Hospital, LMU Munich, Munich, 80336 Germany; 4 Department of Neurology, University Hospital rechts der Isar, School of Medicine, Technical University of Munich, Munich, 81675 Germany; 5 Psychiatrieverbund Oldenburger Land gGmbH, Karl-Jaspers-Klinik, Bad Zwischenahn, 26160 Germany; 6 Department of Psychiatry and Psychotherapy, University Medical Center Göttingen, Göttingen, 37075 Germany; 7 German Center for Neurodegenerative Diseases (DZNE), Göttingen, 37075 Germany; 8 Neurosciences and Signaling Group, Institute of Biomedicine (iBiMED), Department of Medical Sciences, University of Aveiro, Aveiro, 3810-193 Portugal; 9 Department of Psychosomatic Medicine and Psychotherapy, University Medical Center Rostock, Rostock, 18147 Germany; 10 Department of Psychiatry and Psychotherapy, Bezirkskrankenhaus Augsburg, Augsburg, 86156 Germany; 11 Institute for Translational Psychiatry, University of Münster, Münster, 48149 Germany; 12 Department of Psychiatry and Psychotherapeutic Medicine, Research Unit for Bipolar Affective Disorder, Medical University of Graz, Graz, 8036 Austria; 13 Department of Psychiatry and Psychotherapy, University Medical Center Hamburg-Eppendorf, Hamburg, 20246 Germany; 14 Department of Psychiatry, Health North Hospital Group, Bremen, 28102 Germany; 15 Department of Psychiatry and Psychotherapy, Philipps-University Marburg, Marburg, 35039 Germany; 16 Centre for Mind, Brain and Behaviour, University of Marburg, Marburg, 35032 Germany; 17 Department of Psychiatry and Psychotherapy, Agaplesion Diakonieklinikum, Rotenburg, 27356 Germany; 18 International Max Planck Research School for Translational Psychiatry (IMPRS-TP), Max Planck Institute of Psychiatry, Munich, 80804 Germany; 19 Department of Psychiatry, Ruhr University Bochum, LWL University Hospital, Bochum, 44791 Germany; 20 Core-Facility Brainimaging, Faculty of Medicine, University of Marburg, Marburg, Germany; 21 Department of Psychiatry II, Ulm University, Bezirkskrankenhaus Günzburg, Günzburg, 89312 Germany; 22 Clinic for Psychiatry and Psychotherapy, Clinical Center Werra-Meißner, Eschwege, 37269 Germany; 23 Karl-Jaspers Clinic, European Medical School Oldenburg-Groningen, Oldenburg, 26160 Germany; 24 Department of Psychiatry and Psychotherapy, University Tübingen, Tübingen, 72076 Germany; 25 AMEOS Clinical Center Hildesheim, Hildesheim, 31135 Germany; 26 Center for Systems Neuroscience (ZSN), Hannover, 30559 Germany; 27 Department of Psychiatry, Medical School of Hannover, Hannover, 30625 Germany; 28 Department of Psychiatry, University of Münster, Münster, 48149 Germany; 29 Department of Psychiatry, Melbourne Medical School, The University of Melbourne, Melbourne, Australia; 30 The Florey Institute of Neuroscience and Mental Health, The University of Melbourne, Parkville, VIC Australia; 31 Department of Psychiatry and Psychotherapy, Mental Health Institute Berlin, Berlin, 14050 Germany

**Keywords:** Genomics, Psychology

## Abstract

Executive functions are metacognitive capabilities that control and coordinate mental processes. In the transdiagnostic PsyCourse Study, comprising patients of the affective-to-psychotic spectrum and controls, we investigated the genetic basis of the time course of two core executive subfunctions: set-shifting (Trail Making Test, part B (TMT-B)) and updating (Verbal Digit Span backwards) in 1338 genotyped individuals. Time course was assessed with four measurement points, each 6 months apart. Compared to the initial assessment, executive performance improved across diagnostic groups. We performed a genome-wide association study to identify single nucleotide polymorphisms (SNPs) associated with performance change over time by testing for SNP-by-time interactions using linear mixed models. We identified nine genome-wide significant SNPs for TMT-B in strong linkage disequilibrium with each other on chromosome 5. These were associated with decreased performance on the continuous TMT-B score across time. Variant rs150547358 had the lowest *P* value = 7.2 × 10^−10^ with effect estimate beta = 1.16 (95% c.i.: 1.11, 1.22). Implementing data of the FOR2107 consortium (1795 individuals), we replicated these findings for the SNP rs150547358 (*P* value = 0.015), analyzing the difference of the two available measurement points two years apart. In the replication study, rs150547358 exhibited a similar effect estimate beta = 0.85 (95% c.i.: 0.74, 0.97). Our study demonstrates that longitudinally measured phenotypes have the potential to unmask novel associations, adding time as a dimension to the effects of genomics.

## Introduction

The term “executive functions” (EFs) describes a group of higher-level cognitive abilities [1], including the regulation of thoughts and actions in daily life [1, 2]. As humans age, EFs pass different developmental stages, in which great variability is observed both within and between individuals [3, 4]. EFs naturally decline with advanced age [4–6] in a gender-specific manner [7] and diminished EFs are also observed in the longitudinal course of severe mental disorders, such as schizophrenia [8]. In particular, EFs appear to be generally impaired in psychiatric patients suffering from schizophrenia, depression [4], or bipolar disorder [9]. Deficits are also associated, for example with decreased abilities to perform routine tasks [4]. Neurobiologically, EFs are linked intimately to the prefrontal cortex, as exemplified by the famous case of Phineas Gage [10].

There are many definitions of an EF [3], as it represents an umbrella term for multiple cognitive processes [2]. An influential theory of EFs is the “unity and diversity” concept [3, 11] that describes EFs as a “collection of related but separable abilities“ [3]. EFs are differentiated into three latent core skills [3, 4, 11]: (i) set-shifting, allowing an individual to approach tasks flexibly and adjust to new conditions [3, 4], (ii) updating (or working memory), with respect to the monitoring, manipulating, and updating of information [4, 11], and (iii) inhibition, enabling an individual to control behavior, emotions, and responses [4, 11]. In general, EFs rank among the “most heritable psychological traits” [3]. On the behavioral genetic level, a highly heritable latent (common) factor affecting all EF aspects accounted for 99% of the variance common to all three skills [3]. Regarding specific EF components, the heritability estimates of set-shifting assessed by the Trail Making Test (TMT) range from 0.34 to 0.65 [12] and the estimates of updating measured by digit span tests range from 0.27 to 0.62 [12] (these results were obtained in twin studies). Recently, several genome-wide association studies (GWASs) on EFs have been undertaken [13–18]; however, genome-wide significance was not attained [2, 12]. Moreover, the genetic basis of variation over time is yet to be elucidated [19].

Here, we performed two longitudinal GWASs for the set-shifting and updating EF abilities assessed by the Trail Making Test, part B (TMT-B) and the Verbal Digit Span backwards (VDS-B), respectively, to identify genetic variation associated with the course of EFs across time. We used a linear mixed model (LMM) to model the dependence structure of the longitudinal PsyCourse Study [20] with four measurements across time. To validate our findings, we also performed a replication study using data from the FOR2107 consortium [21], which assessed two measurements over time.

## Materials and methods

### Discovery sample: PsyCourse Study

The PsyCourse Study is a multicenter longitudinal study that combines multilevel omics and longitudinal data [20]. We included 1338 genotyped individuals (dataset version 3.0) recruited in different centers in Germany and Austria, comprising patients from the affective-to-psychotic spectrum (377 bipolar I disorder, 100 bipolar II disorder, 420 schizophrenia, 95 schizoaffective disorder, 6 brief psychotic disorder, 9 schizophreniform disorder, and 73 with recurrent depression) and 258 psychiatrically healthy controls. The study protocol was approved by the respective ethics committee for each study center and was carried out following the rules of the Declaration of Helsinki of 1975, revised in 2008 (see ref. [20]). All study participants provided written consent [20]. The patients were diagnosed using parts of the Structured Clinical Interview for DSM (SCID-I) and were classified according to the Diagnostic and Statistical Manual of Mental Disorders, Fourth Edition (DSM-IV) criteria. The patients were broadly differentiated in patients with predominantly affective symptoms (550 “affective”, with recurrent depression, bipolar I and II disorders) and patients with predominantly psychotic symptoms (530, “psychotic”, with schizophrenia, schizoaffective, brief psychotic and schizophreniform disorder) [20]. Deep phenotyping was performed during four visits, each ~6 months apart (see ref. [20]), thus corresponding to time *t* of the longitudinal course.

Set-shifting and updating were assessed with the Trail Making Test, part B (TMT-B) [22] and the Verbal Digit Span backwards (VDS-B) [23], respectively. The TMT-B requires an individual to connect numbers (numbers: 1–26) and letters of the alphabet in ascending alternating order. The test score was the time (in seconds (s)) needed to finish this exercise. As recommended by [24] participants with a time >300 s were set to 300 s. VDS-B measures the updating ability. Here, a trained interviewer verbally presented up to seven pairs of number sequences with increasing length, and the study participant was requested to repeat each sequence in backwards order, receiving a point score for each correctly repeated sequence. The maximum possible score for each sequence pair was 2. The process was terminated when an individual failed to repeat correctly both of the sequences in a pair of given length. The test score was the sum of all correctly repeated sequence pairs (range: 0–14).

### Replication sample: FOR2107 consortium

To perform the replication study, we used data from the research consortium FOR2107 [21], a longitudinal cohort with two centers, Marburg and Münster (Germany), in which deep phenotyping was performed twice ~2 years apart [21]. In our analyses, we used a sample comprising 1795 individuals with genotype data available divided into five different diagnostic groups (851 affective: 107 bipolar disorder and 744 depression, 112 psychotic: 68 schizophrenia and 44 schizoaffective disorder, and 832 healthy controls). The participants were classified into the same three broad diagnostic groups (affective, psychotic, and controls) as in the discovery sample. Set-shifting was assessed by the TMT-B. In this cohort, participants with a time >180 s were excluded. For updating, we used the Letter–Number-Sequencing Test (LNST) as a substitute for the VDS-B. Here, a trained interviewer verbally presented an increasing sequence of letters and numbers, which the participant was requested to repeat, starting with the numbers in ascending order and ending with the letters in alphabetical order. The test was terminated when the individual repeated the same sequence incorrectly four times. The sum of the correctly repeated sequences was the test score, with a maximum of 24.

### Genotyping and imputation

#### Discovery sample

The Illumina Infinium PsychArray (Illumina, USA) was used for genotyping purposes [20]. Genotypes were imputed with SHAPEIT2/IMPUTE2 using the 1000 Genomes Project Phase 3 data as a reference panel. Quality control (QC) was performed according to standard procedures, as described previously [25] (details Supplementary List 1) and poorly imputed genetic variants (INFO < 0.8) were excluded [20]. We included ~8.2 million SNPs with minor allele frequency (MAF) ≥ 0.01 in our analysis. Ancestry principal components (PCs) were computed with PLINK v1.9 [26] (http://pngu.mgh.harvard.edu/).

#### Replication sample

To replicate genome-wide significant SNPs of the discovery sample, we analyzed the genotypes of these nine significant SNPs (SNP_R_). We additionally analyzed 187 suggestive SNPs (SNP_NR_) with a *P* value ≤1 × 10^−5^ in the discovery sample (99 for TMT-B, 88 for VDS-B/LNST) in an exploratory analysis. For the QC in the replication sample, please refer to Supplementary List 2.

### Statistical analysis

We performed regression analysis, log-transforming the TMT-B values (lgTMT-B) to fulfill the linear mixed model requirement of normally distributed errors. We present effect estimates with 95% confidence intervals (c.i.s) transformed back to the original scale. Furthermore, we investigated missing data patterns across visits and diagnoses for violation of a missing-at-random (MAR) mechanism [27]. We computed the mean and standard deviation (s.d.) of EFs per visit and diagnostic group, testing for differences in means between diagnostic groups at each visit. For the discovery sample, we fitted LMMs to the longitudinal time course of lgTMT-B and VDS-B, investigating each phenotype first without the SNP terms, and subsequently including them. For each SNP, the fitted model for individual *i* at visit/time *t*_*ij*_ with *j* = 1, 2, 3, 4 was as follows:

$$\begin{array}{l}Y_{ij} = \beta _0 + \beta _1t_{ij} + \beta _2age_i + \beta _3gender_i + \beta _4diagnosis_i + \mathop {\sum }\limits_{k = 1}^5 \beta _{4 + k}PC_{ik} + \\ b_{0i} + b_{1i}t_{ij} + c_icenter_i + \beta _{10}SNP_i + \beta _{11}SNP_i \ast t_{ij} + \varepsilon _{ij}\end{array}$$

The LMM adjusted for *age*_*i*_*, gender*_*i*_*, diagnosis*_*i*_, *PC*_*ik*_, i.e., age at visit 1, gender, diagnostic group (affective, psychotic, or control), and the top five PCs, for each individual *i*, the latter to correct for population stratification. We allowed for random intercepts and slopes *b*_0*i*_*,b*_1*i*_ of the trajectories and a random center effect.

For the respective SNP under consideration, we integrated the main effect (*SNP*_*i*_) and the SNP-by-time interaction (*SNP*_i_*t_*ij*_), where the latter is tested (two-sided) for the influence of the SNP on the longitudinal course (see ref. [28]). The interaction term consisting of SNP × diagnosis × time has not been investigated due to the limited sample size. We assumed an additive genetic model with each considered SNP in dosage format. We set the genome-wide significance level to 5 × 10^−8^, yielding replication SNPs (SNP_R_), and set the level for suggestive significance to 1 × 10^−5^ for SNPs to be further explored (SNP_NR_, not to be replicated). For the replication sample, we separately determined linkage disequilibrium (LD) blocks with *r*^2^ > 0.8 for both SNP sets, correcting for multiple testing by dividing 5% by the number of LD blocks for the SNP set [29]. In the end, the SNP_R_ were contained in a single LD block, so the significance level for replication could be set to 5%. The significance levels for the exploratory analysis of the SNP_NR_ were set to 0.05/24 = 0.0021 for lgTMT-B and 0.05/12 = 0.0042 for VDS-B/LNST, respectively.

For the SNP analysis in the replication sample, we analyzed the difference (diff) of lgTMT-B (LNST) between the visits as outcome and SNP, age, gender, diagnosis, and PC’s as covariates. We applied the difference model, as the LMM above contained too many parameters for the replication sample with only two measurements (in total: 613 individuals) and incomplete data resulting in low statistical power (data not shown; two-sided test). Here, the SNP effect may be interpreted as the difference between the average change between the genotypes, especially since SNP_R_ displayed only two genotypes.

We computed LD and haplotypes for Europeans with LDlink [30] and created a regional plot with gene identification using Locus-Zoom [31]. Finally, the average longitudinal course over time per genotype along with 95% c.i. is displayed for the top SNP.

All statistical analyses were performed with R, version 3.5.1 (https://www.r-project.org/). The LMM was fitted with the R package lme4 [32] and *P* values were computed using the Satterthwaite approximation of the lmerTest package [33, 34].

## Results

### Behavioral characteristics of the EFs

#### Discovery sample

**Fig. 1:**
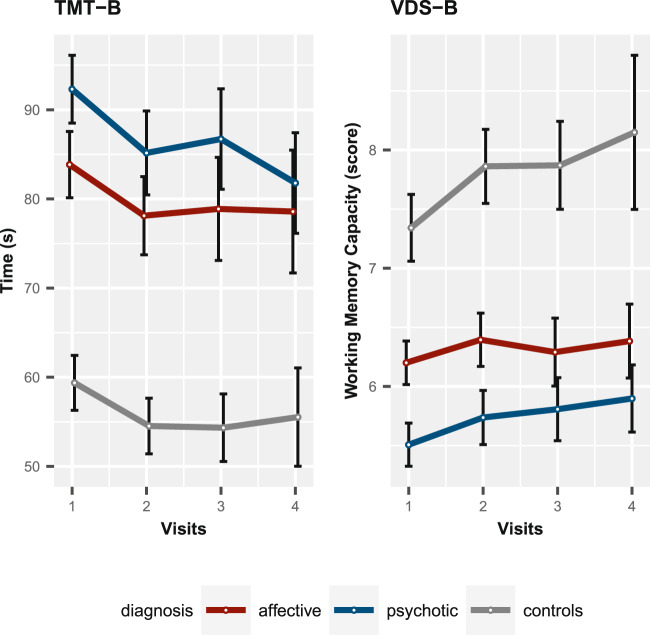
Longitudinal course of TMT-B score (time in seconds, left) and VDS-B score (working memory capacity, right) for each diagnostic group in the discovery sample. Displayed are means with 95% confidence interval for each visit 1, 2, 3, 4, ~6 months apart.

In comparison with controls, the disease groups were slightly older on average (Table 1). A total of 1272 (1297) individuals had at least one TMT-B (VDS-B) measurement, demonstrating a similar decrease of available data in each diagnostic group (Table 2). Missing value patterns did not hint at any violation of a missing-at-random (MAR) assumption (data not shown). Figure 1 illustrates the mean longitudinal course of TMT-B (left) and VDS-B (right) for each diagnostic group with 95% c.i.s; controls differed significantly from patients (see Fig. 1, c.i.s). Generally, executive performance increased over time, with differences between affective and psychotic patients decreasing over time. An improvement in the respective EF performance is reflected by a decreased TMT-B score for set-shifting and an increased VDS-B score for updating. The individual trajectories were highly variable (Supplementary Fig. 1). The mean difference between diagnostic groups was significant at each visit when adjusting for age and gender (see Table 1). Table 3 displays the time effect estimates in the LMM for each phenotype without SNP stratified by diagnostic group. For lgTMT-B, the time effect within each diagnostic group is highly significant and similar across groups. For VDS-B, the time effects for the two patient groups are similar, very small, and only nominally significant in the psychotic group, but larger and highly significant for controls.

**Table 1:** Characteristics at visit 1 in discovery sample and replication sample by diagnostic group.

Study sample	Phenotypes	Diagnostic groups mean (s.d.) or percentage (%)			Group difference
		Affective	Psychotic	Controls	*P* value
Discovery sample	Age	44.6 (13.4)	41.1 (12.1)	37.1 (15.6)	–
	Females	49.8 %	39.6 %	58.1 %	–
	TMT-B	83.9 (42.6)	92.3 (41.3)	59.4 (25.1)	<2 × 10^−16^
	VDS-B	6.2 (2.1)	5.5 (2.0)	7.3 (2.9)	<2 × 10^−16^
Replication sample	Age	37.6 (13.4)	38.4 (11.3)	34.1 (12.6)	–
	Females	63.9 %	44.6 %	63.0 %	–
	TMT-B	57.7 (23.9)	73.6 (30.9)	48.8 (18.6)	<2 × 10^−16^
	LNST	15.7 (3.3)	13.4 (3.5)	16.8 (3.2)	<2 × 10^−16^

The proportion of females (%), means of age (years), TMT-B, and VDS-B/LNST with standard deviation (s.d.).

We tested for differences in means between the diagnostic groups for lgTMT-B and VDS-B. Results are only displayed for visit 1 as results for the other visits proved to be similar.

**Table 2:** Available data of TMT-B and VDS-B per visit for the discovery sample.

EF core skill	Diagnostic groups											
	Affective				Psychotic				Controls			
Visit (t)	1	2	3	4	1	2	3	4	1	2	3	4
TMT-B	506 (92%)	315 (57%)	234 (43%)	182 (33%)	456 (86%)	295 (56%)	252 (46%)	227 (48%)	258 (100%)	225 (82%)	178 (69%)	57 (22%)
VDS-B	503 (92%)	324 (59%)	234 (43%)	185 (34%)	479 (90%)	320 (60%)	265 (50%)	236 (45%)	257 (99.6%)	225 (87%)	178 (69%)	60 (23%)

Absolute numbers and percent of group total within the diagnostic group with 550 affective individuals, 530 psychotic individuals, and 258 controls.

**Table 3:** Results of the LMM of the discovery sample to test the time effect on lgTMT-B and VDS-B within each diagnostic group.

EF core skill		TMT-B			VDS-B		
Diagnostic groups	Time effect (t)	*β*	95% c.i.	*P* value	*β*	95% c.i.	*P* value
Affective		0.957	0.94, 0.97	9.8 × 10^−09^	0.076	0, 0.15	0.053
Psychotic		0.950	0.94, 0.96	<2 × 10^−16^	0.086	0.02, 0.15	0.011
Controls		0.947	0.93, 0.96	6.1 × 10^−11^	0.288	0.17, 0.41	2.7 × 10^−06^

The effect estimates β of lgTMT-B are transformed back to their original scale.

#### Replication sample

We analyzed 1795 genotyped individuals with at least one TMT-B and LNST measurement (we deleted data for one individual who had a value larger than the maximum score of 24). Phenotypes were measured at both visits for 34.2%. The means of the diagnostic groups at each visit were significantly different (Table 1) during which the controls had again the best EF abilities, followed by affective and then psychotic individuals (Supplementary Fig. 2).

### GWAS of the discovery sample

**Fig. 2:**
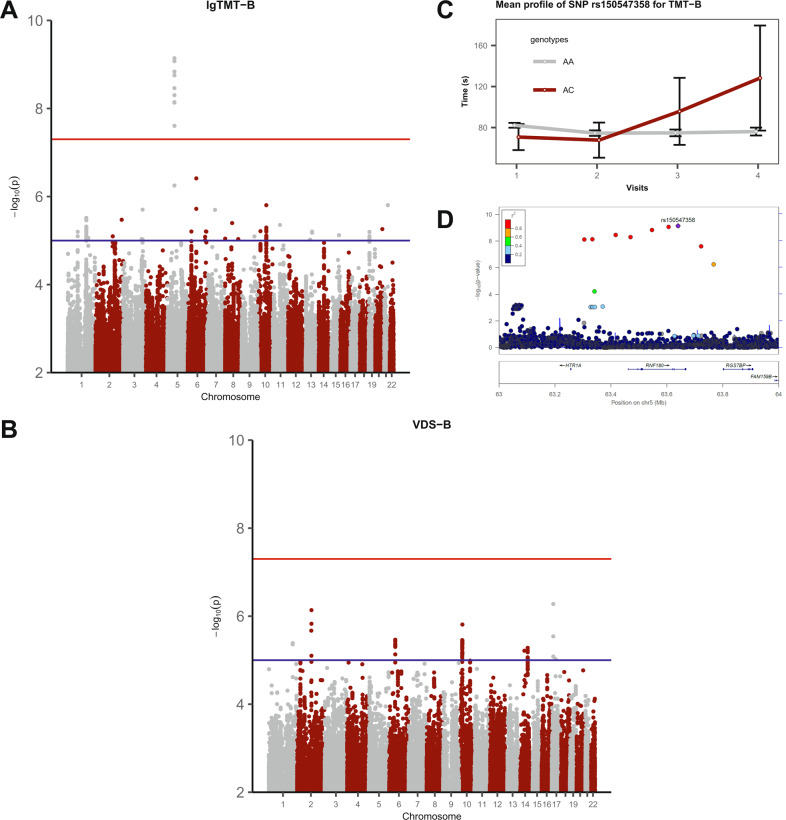
Results of the genome-wide association studies of the discovery sample. **A** Manhattan plot of the GWAS of lgTMT-B in the discovery sample. The lines in (**A**) and (**B**) indicate the thresholds for the genome-wide significance of 5 × 10^−8^ (red) and for suggestive SNPs (blue, *P* ≤ 1 × 10^−5^). **B** Manhattan plot of the GWAS of VDS-B in the discovery sample. **C** Mean profile of TMT-B by the top SNP rs150547358 genotypes for the discovery sample (1039 AA, 28 AC, 0 CC) with the 95% confidence intervals. **D** GWAS regional Manhattan plot of chromosome 5 for lgTMT-B of the discovery sample. Colors indicate the LD values (*r*^2^) of SNPs with rs150547358 (in purple).

The QQ-plot (Supplementary Fig. 3) demonstrates that the genomic inflation factor was *λ* = 1.0034 for lgTMT-B and *λ* = 0.9999 for VDS-B, hence not indicating any inflation. As illustrated on the Manhattan plots (lgTMT-B Fig. 2A, VDS-B Fig. 2B) for the SNP-by-time interaction in the LMM, we identified nine genome-wide significant SNPs on chromosome 5 (all imputed) in one LD block (*r*^2^ > 0.85) for lgTMT-B, and none for VDS-B. For lgTMT-B, 99 SNPs were suggestive, for VDS-B 88.

For the nine genome-wide significant SNPs of the GWAS, Supplementary Table 1 displays estimates for the effect of the SNP-by-time interaction on lgTMT with 95% c.i. and *P* values. The top SNP rs150547358 (*P* value = 7.2 × 10^−10^) had an effect of 1.16 (95% c.i. 1.11–1.22) seconds per measurement (spm) in the discovery sample on the original TMT-B scale. We present the mean plot for the top SNP in Fig. 2C, where the TMT-B score increases over time for heterozygotes with risk allele “C”. Figure 2D displays the regional Manhattan plot with three genes in or near the nine significant SNPs. Four of them, including rs150547358, are located in an intron region of ring finger protein 180 (RNF180) (Supplementary Table 1). Other genes located nearby are regulator of G protein signaling 7 binding protein (RGS7BP) and 5-hydroxytryptamine receptor 1A (HTR1A), but neither contained any of the nine SNPs. For the SNP main effect, also included in the model, we did not observe any genome-wide significant SNPs (Supplementary Fig. 4; *P* < 5 × 10^−8^).

### Difference analysis of the replication sample

The analysis of the differences also identified the top SNP, rs150547358, as significant (*P* = 0.015), and thus replicated this GWAS-significant LD block. The effect estimate for the top SNP was 0.85 (95% c.i. 0.74–0.97) on the original scale and the highest effect size in the scale of the analysis (greatest negative effect). The estimates for the other SNPs were slightly larger when transformed back to the original scale and also positive (see Supplementary Table 1 for the summary).

Exploratory analysis of the GWAS-suggestive SNP_NR_ in the replication sample yielded no significant results after multiple testing corrections for either phenotype (Supplementary Fig. 5).

## Discussion

We performed a GWAS on the longitudinal course of EFs and detected nine SNPs within the same LD block associated with change over a relatively short period of time (∼1.5 years) in the EF core skill set-shifting. Importantly, we were able to replicate a significant result for this LD block in an independent sample, which was observed in a heterogeneous population including controls and different psychiatric disorders of the affective-to-psychotic spectrum across age groups. Analysis of TMT-B performance of C-allele carriers, in contrast to the AA genotype, revealed a pronounced slowing over time.

Recently, the analysis of longitudinal data has come to the fore in genetic research. Multiple methods have been developed to perform GWAS with longitudinal data [35–40] for binary as well as continuous phenotypes. These analysis methods are mostly applied to analyze long-term developments of the investigated phenotypes [41, 42], as most data comprise multiple measurements over a relatively long period of time. These longitudinal studies often detect group effects [8] based on age or baseline cognitive functions, for example. To date, short-term variability, for example with respect to the longitudinal course of schizophrenia has been found as reviewed [8], but without considering a potential genetic effect. In our longitudinal GWAS, we enter uncharted territory as we study short-term courses of cognitive phenotypes in relation to the genetic background. The discovery sample, the PsyCourse Study, is unique in this sense, as it assesses the phenotypes multiple times in a very heterogeneous sample over a relatively short period of time (18 months). Here, the main interest is the observation of short-term changes specific to a phenotype, such as EF skills, and the use of newly identified characteristics to detect genotype–phenotype associations. The genetic variants found in this study may, if further replicated, be used to improve clinical evaluation of the longitudinal course of EF skills. Knowledge of the genetic status of a patient may, in the future, enhance the interpretation of the course of EF abilities e.g., during psychiatric treatment. Moreover, special training programs could support patients with a known genetic disposition to lack improvement over time. To our knowledge, no other study has performed such analyses to date.

### Behavioral results

Prior to our GWAS, we studied the short-term courses of changes in cognitive abilities, focusing on the differences between the diagnostic groups considered. In the discovery sample, we observed an identical pattern for both phenotypes: psychotic individuals demonstrated the lowest EF abilities, followed by those with affective disorders and then the control individuals. This greater EF impairment in psychotic individuals compared to controls is well-documented, as exemplified by [43]. However, regarding the impairment difference between bipolar (affective) and schizophrenic (psychotic) patients, there are various studies [43–48] analyzing these differences. The hypothesis exists that bipolar patients demonstrate less severe impairment in comparison to schizophrenic patients [49]. Some studies [44, 46, 48] lend their support to this hypothesis, though not always statistically significant, whereas others detected similar levels of impairment in symptomatic patients [45, 47]. In our analysis, we observed a statistically significant difference between affective and psychotic individuals at visit 1 but detected a decline in these discrepancies over time. The abilities of these two diagnostic groups converged with patients from the psychotic group displaying an improvement in their skills and patients from the affective group presenting a more constant course. Documentation of the EF convergence is only possible thanks to the longitudinal design of the discovery sample and represents a great advantage of this study design.

Owing to the slightly different age structure of the two study samples, with the discovery sample being minimally older on average at visit 1, we further observed the impact of age reflected by the minimally lower average test score. That is, the discovery sample had lower VDS and greater TMT-B scores than the replication sample. The TMT-B mean scores may also be influenced further by the different cutoff thresholds of 300 s in the discovery sample and 180 s for the replication sample.

### Genome-wide association studies

To our knowledge, the LD block comprising the nine SNPs we detected for the set-shifting ability has been not identified in any GWAS before. These SNPs are part of two common haplotypes, that is, 97.7% carry the haplotype consisting of the major alleles and 1.7% have the rare haplotype with only minor alleles in European populations [30]. However, we did not observe different allelic distributions between the three diagnostic groups (Supplementary Table 2). We displayed the longitudinal course for the two genotypes “AC” and “AA” of the top SNP rs150547358, observing a steady increase in the TMT-B score for “AC” and an almost unchanging course for “AA”. Consequently, the minor allele C was associated with a decline in the set-shifting ability of ~5 s over a period of 18 months for AC with a large c.i. at the last visit owing to the small number of available heterozygous individuals. This result reflects a relatively high decrease in the ability over this short period. Furthermore, it portrays a highly interesting observation, which is further underpinned when we consider the genetic region of the nine SNPs. Variant rs150547358, the significantly replicated SNP, is one of four associated SNPs directly located in the ring finger protein 180 (RNF180) gene on chromosome 5q12.3. It is an E3 ubiquitin-protein ligase [50], whose product is involved in protein modification. RNF180 is associated with the regulation of monoamine levels in different brain regions, for example, the prefrontal cortex (PFC) in RNF180 knockout mice [51]. The PFC is a critical part of the frontal lobe in the development of EFs [4, 52]. Another gene located in the nearby region, HTR1A (5-hydroxytryptamine receptor 1A), is an important receptor of serotonin (5-HT) also essential to the prefrontal lobe. More importantly, HTR1A is an autoreceptor, located on the cell bodies of serotonin-synthesizing neurons of the brainstem dorsal raphe nucleus, helping to maintain homeostasis in serotonergic function [53]. Furthermore, a genetic polymorphism in the 5-HT system has previously been implicated in EF performance [12].

In an additional exploratory gene-set analysis performed with MAGMA v1.06 as a part of the FUMA pipeline (https://fuma.ctglab.nl/) [54], we did not receive significant (Bonferroni-corrected *P* values ≤0.05) pathways for either phenotype.

Our results are a first step in the direction of understanding the molecular genetic influences on the longitudinal course of EFs. We were unable to consider the third core ability, inhibition, which also plays an important role for EF, because we could not fulfill a specific assessment requirement resulting from the multicenter and interview-based structure of the discovery sample [20]. Many unknown factors remain, such as the genetic aspects due to the correlation of the different EF abilities, as we only concentrated on individual EF core skills in two separate analyses. According to the “unity but diversity” concept [11] that also concerns the genetic underpinnings of the EFs, a genetic study of a latent common factor needs to follow. Further, we need to acknowledge the problem of missing data which is a great challenge in longitudinal studies as presented in our samples. Here, selecting the correct analysis method, e.g., linear mixed models are imported but generally, more longitudinal studies with multiple time points and greater sample sizes will be required to unmask further time and genomics interactions [19].

## Supplementary information

Supplementary Information

## Data Availability

R code and data will be available upon reasonable request by the authors. The summary statistics of our analysis will be published in the GWAS Catalog (https://www.ebi.ac.uk/gwas/).

## References

[1] Friedman NP, Miyake A, Altamirano LJ, Corley RP, Young SE, Rhea SA (2016). Stability and change in executive function abilities from late adolescence to early adulthood: a longitudinal twin study. Developmental Psychol..

[2] Barnes JJM, Dean AJ, Nandam LS, O’Connell RG, Bellgrov MA (2011). The molecular genetics of executive function: role of monoamine system genes. Biol Psychiatry..

[3] Friedman NP, Miyake A, Young SE, DeFries JC, Corley RP, Hewitt JK (2008). Individual differences in executive functions are almost entirely genetic in origin. J Exp Psychol: General..

[4] Diamond A (2013). Executive functions. Annu Rev Psychol..

[5] Best JR, Miller PH, Jones LL (2009). Executive functions after age 5: changes and correlates. Developmental Rev.

[6] West R, Wiebe SA, Karbach J (2017). Aging and the neural correlates of executive function. Executive function.

[7] van Hooren SA, Valentijn AM, Bosma H, Ponds RW, van Boxtel MP, Jolles J (2007). Cognitive functioning in healthy older adults aged 64-81: a cohort study into the effects of age, sex, and education. Aging Neuropsychol Cognition..

[8] Heilbronner U, Samara M, Leucht S, Falkai P, Schulze TG. The longitudinal course of schizophrenia across the lifespan. Harv Rev Psychiatry. 2016;24:118–28. 10.1097/HRP.0000000000000092 PMC5079232 26954596

[9] Martínez-Arán A, Vieta E, Colom F, Torrent C, Sánchez-Moreno J, Reinares M (2004). Cognitive impairment in euthymic bipolar patients: implications for clinical and functional outcome. Bipolar Disord..

[10] Ratiu P, Talos IF (2004). The tale of phineas gage, digitally remastered. N Engl J Med..

[11] Miyake A, Friedman NP, Emerson MJ, Witzki AH, Howerter A, Wager TD (2000). The unity and diversity of executive functions and their contributions to complex “frontal lobe” tasks: a latent variable analysis. Cogn Psychol..

[12] Li JJ, Roberts DK. Genetic influences on executive functions across the life span. In: Wiebe SA, Karbach J, (eds.) Executive function. New York: Routledge; 2017. p. 106–23.

[13] Luciano M, Hansell NK, Lahti J, Davies G, Medland SE, Räikkönen K (2011). Whole genome association scan for genetic polymorphisms influencing information processing speed. Biol Psychol..

[14] Seshadri S, DeStefano AL, Au R, Massaro JM, Beiser AS, Kelly-Hayes M, et al. Genetic correlates of brain aging on MRI and cognitive test measures: a genome-wide association and linkage analysis in the Framingham study. BMC Med Genet. 2007;8:1–14. 10.1186/1471-2350-8-S1-S15 PMC1995608 17903297

[15] Cirulli ET, Kasperaviciūte D, Attix DK, Need AC, Ge D, Gibson G (2010). Common genetic variation and performance on standardized cognitive tests. Eur J Hum Genet..

[16] Need AC, Attix DK, McEvoy JM, Cirulli ET, Linney KL, Hunt P (2009). A genome-wide study of common SNPs and CNVs in cognitive performance in the CANTB. Hum Mol Genet..

[17] Malone SM, Vaidyanathan U, Basu S, Miller MB, McGue M, Iacono WG (2014). Heritability and molecular-genetic basis of the P3 event-related brain potential: a genome-wide association study. Psychophysiology..

[18] LeBlanc M, Kulle B, Sundet K, Agartz I, Melle I, Djurovic S (2012). Genome-wide study identifies PTPRO and WDR72 and FOXQ1-SUMO1P1 interaction associated with neurocognitive function. J Psychiatr Res..

[19] Boyce WT, Sokolowski MB, Robinson GE (2020). Genes and environments, development and time. Proc Natl Acad Sci USA..

[20] Budde M, Anderson-Schmidt H, Gade K, Reich-Erkelenz D, Adorjan K, Kalman JL (2018). A longitudinal approach to biological psychiatric research: the PsyCourse study. Am J Med Genet Part B: Neuropsychiatr Genet..

[21] Kircher T, Wöhr M, Nenadic I, Schwarting R, Schratt G, Alferink J (2018). Neurobiology of the major psychoses: a translational perspective on brain structure and function—the FOR2107 consortium. Eur Arch Psychiatry Clin Neurosci..

[22] Bowie CR, Harvey PD (2006). Administration and interpretation of the trail making test. Nat Protoc..

[23] Hilbert S, Nakagawa TT, Puci P, Zech A, Bühner M (2015). The digit span backwards task. Eur J Psychologcal Assess..

[24] Strauss E, Sherman EMS, Spreen O. A compendium of neuropsychological tests—administration, norms, and commentary. New York: Oxford University Press; 2006.

[25] Andlauer TF, Buck D, Antony G, Bayas A, Bechmann L, Berthele A (2016). Novel multiple sclerosis susceptibility loci implicated in epigenetic regulation. Sci Adv..

[26] Purcell S, Neale B, Todd-Brown K, Thomas L, Ferreira MA, Bender D (2007). PLINK: a tool set for whole-genome association and population-based linkage analyses. Am J Hum Genet..

[27] Molenbergh G, Verbeke, G. Linear mixed models for longitudinal data. Berlin, Heidelberg: Springer; 2000.

[28] Sikorska K, Rivadeneira F, Groenen PJF, Hofman A, Uitterlinden AG, Eilers PHC (2012). Fast linear mixed model computations for genome-wide association studies with longitudinal data. Stat. Med..

[29] Duggal P, Gillanders EM, Holmes TN, Bailey-Wilson JE (2008). Establishing an adjusted p-value threshold to control the family-wide type 1 error in genome wide association studies. BMC Genomics..

[30] Machiela MJ, Chanock SJ (2015). LDlink: a web-based application for exploring population-specific haplotype structure and linking correlated alleles of possible functional variants. Bioinformatics..

[31] Pruim RJ, Welch RP, Sanna S, Teslovich TM, Chines PS, Gliedt TP (2010). LocusZoom: regional visualization of genome-wide association scan results. Bioinformatics..

[32] Bates D, Mächler M, Bolker B, Walker S (2015). Fitting linear mixed-effects models using lme4. J Stat Softw..

[33] Kuznetsova A, Brockhoff PB, Christensen RHB. lmerTest package: tests in linear mixed effects models. J Stat Softw. 2017;82:1–26.

[34] Luke SG (2016). Evaluating significance in linear mixed-effects models in R. Behav Res Methods..

[35] Sikorska K, Lesaffre E, Groenen PJF, Rivadeneira F, Eilers PHC. Genome-wide analysis of large-scale longitudinal outcomes using penalization GALLOP algorithm. Sci Rep. 2018;8:1–8. 10.1038/s41598-018-24578-7 PMC5931565 29717146

[36] Sikorska K, Montazeri NM, Uitterlinden A, Rivadeneira F, Eilers PH, Lesaffre E (2015). GWAS with longitudinal phenotypes: performance of approximate procedures. Eur J Hum Genet..

[37] Wu W, Wang Z, Xu K, Zhang X, Amei A, Gelernter J (2019). Retrospective association analysis of longitudinal binary traits identifies important loci and pathways in cocaine use. Genetics..

[38] Rudra P, Broadaway KA, Ware EB, Jhun MA, Bielak LF, Zhao W (2018). Testing cross-phenotype effects of rare variants in longitudinal studies of complex traits. Genet Epidemiol..

[39] Ning C, Wang D, Zhou L, Wei J, Liu Y, Kang H (2019). Efficient multivariate analysis algorithms for longitudinal genome-wide association studies. Bioinformatics..

[40] Lee Y, Park S, Moon S, Lee J, Elston RC, Lee W (2014). On the analysis of a repeated measure design in genome-wide association analysis. Int J Environ Res Public Health..

[41] Adkins DE, Clark SL, Copeland WE, Kennedy M, Conway K, Angold A (2015). Genome-wide meta-analysis of longitudinal alcohol consumption across youth and early adulthood. Twin Res Hum Genet..

[42] Tang W, Kowgier M, Loth DW, Soler Artigas M, Joubert BR, Hodge E (2014). Large-scale genome-wide association studies and meta-analyses of longitudinal change in adult lung function. PLoS ONE..

[43] Wobrock T, Ecker UK, Scherk H, Schneider-Axmann T, Falkai P, Gruber O (2009). Cognitive impairment of executive function as a core symptom of schizophrenia. World J Biol Psychiatry..

[44] Szoke A, Meary A, Trandafir A, Bellivier F, Roy I, Schurhoff F (2008). Executive deficits in psychotic and bipolar disorders - Implications for our understanding of schizoaffective disorder. Eur Psychiatry..

[45] Amann B, Gomar JJ, Ortiz-Gil J, McKenna P, Sans-Sansa B, Sarró S (2012). Executive dysfunction and memory impairment in schizoaffective disorder: a comparison with bipolar disorder, schizophrenia and healthy controls. Psychological Med..

[46] Hill SK, Reilly JL, Keefe RS, Gold JM, Bishop JR, Gershon ES (2013). Neuropsychological Impairments in schizophrenia and psychotic bipolar disorder: findings from the bipolar-schizophrenia network on intermediate phenotypes (B-SNIP) study. Am J Psychiatry..

[47] Lewandowski KE, Cohen BM, Keshavan MS, Öngür D (2011). Relationship of neurocognitive deficits to diagnosis and symptoms across affective and non-affective psychoses. Schizophrenia Res..

[48] Reichenberg A, Harvey PD, Bowie CR, Mojtabai R, Rabinowitz J, Heaton RK (2008). Neuropsychological function and dysfunction in schizophrenia and psychotic affective disorders. Schizophrenia Bull..

[49] Lynham AJ, Hubbard L, Tansey KE, Hamshere ML, Legge SE, Owen MJ (2018). Examining cognition across the bipolar/schizophrenia diagnostic spectrum. J Psychiatry Neurosci..

[50] Ogawa M, Mizugishi K, Ishiguro A, Koyabu Y, Imai Y, Takahashi R (2008). Rines/RNF180, a novel RING finger gene-encoded product, is a membrane-bound ubiquitin ligase. Genes Cells..

[51] Kabayama M, Sakoori K, Yamada K, Ornthanalai VG, Ota M, Morimura N (2013). Rines E3 ubiquitin ligase regulates MAO-A levels and emotional responses. J Neurosci..

[52] Best JR, Miller PH (2010). A developmental perspective on executive function. Child Dev..

[53] McDevitt RA, Neumaier F (2011). Regulation of dorsal raphe nucleus function by serotonin autoreceptors: a behavioral perspective. J Chem Neuroanat..

[54] Watanabe K, Taskesen E, van Bochoven A, Posthuma D. Functional mapping and annotation of genetic associations with FUMA. Nat. Commun. 2017;8:1–11. 10.1038/s41467-017-01261-5 PMC5705698 29184056

