# Supplementary material for: A genome-wide association study of the longitudinal course of executive functions
Source: Transl Psychiatry. 2021 Jul 10;11:386. doi: 10.1038/s41398-021-01510-8 (PMC8272719; doi:10.1038/s41398-021-01510-8)
Supplement: Supplementary file 1 — Supplementary Information [file 41398_2021_1510_MOESM1_ESM.docx]

Supplementary information

**Supplementary List 1.**

For the **PsyCourse** study the following quality control steps were performed.

Sequence of Quality Control steps:

1. Removal of SNPs with call rates **<98%** or a MAF **<1%**
2. Removal of individuals with genotyping rates **<98%**
3. Removal of gender mismatches
4. Removal of genetic duplicates
5. Removal of cryptic relatives with $\hat{\boldsymbol{\pi}}$ **≥ 12.5**
6. Removal of genetic outliers with a distance from the mean of >**4** SD in the **first eight** MDS components
7. Removal of individuals with a deviation of the autosomal or X-chromosomal heterozygosity from the mean >**4** SD
8. Removal of non-autosomal variants
9. Removal of SNPs with call rates **<98%** or a MAF **<1%** or Hardy-Weinberg Equilibrium (HWE) test p-values **<1×10-6**
10. Removal of A/T and G/C SNPs
11. Update of variant IDs and positions to the IDs and positions in the 1000 Genomes Phase 3 reference panel
12. Alignment of alleles to the reference panel
13. Removal of duplicated variants and variants not present in the reference panel

Imputation was conducted using SHAPEIT2 (https://mathgen.stats.ox.ac.uk/genetics_software/shapeit/shapeit.html) (1) and IMPUTE2 (http://mathgen.stats.ox.ac.uk/impute/impute_v2.html) (2,3) using the 1000 Genomes Phase 3 as reference panel. Genetic marker with a poor imputation quality (INFO < 0.8) were excluded (4).

**Supplementary List 2.**

**FOR2107**

# Genotyping, quality control, and imputation

Genotyping was conducted using the Infinium PsychArray BeadChip, as described previously (5). The quality control (QC) of genetic data was conducted in PLINK v1.90b6.10 (6) and R v3.5.2, as described previously (7). Pre-imputation QC of genotype data consisted of the following steps:

1. Removal of SNPs with call rates <98% or a minor allele frequency (MAF) <1%
2. Removal of individuals with genotyping rates <98%
3. Removal of sex mismatches
4. Removal of genetic duplicates
5. Removal of cryptic relatives with pi-hat≥12.5
6. Removal of genetic outliers with a distance from the mean of >4 SD in the first eight multidimensional scaling (MDS) ancestry components
7. Removal of individuals with a deviation of the autosomal or X-chromosomal heterozygosity from the mean >4 SD
8. Removal of non-autosomal variants
9. Removal of SNPs with call rates <98% or a MAF <1% or Hardy-Weinberg Equilibrium (HWE) test *p*-values <1×10^-6^
10. Removal of A/T and G/C SNPs
11. Update of variant IDs and positions to the IDs and positions in the 1000 Genomes Phase 3 reference panel
12. Alignment of alleles to the reference panel
13. Removal of duplicated variants and variants not present in the reference panel

For the calculation of ancestry components (used to determine genetic outliers and as covariates in the analyses), pre-imputation genotype data were used. Additional variant filtering steps were removal of variants with a MAF <0.05 or HWE *p*‑value <10^‑3^; removal of variants mapping to the extended MHC region (chromosome 6, 25-35 Mbp) or to a typical inversion site on chromosome 8 (7‑13 Mbp); linkage disequilibrium (LD) pruning (command --indep-pairwise 200 100 0.2). Next, the pairwise identity-by-state (IBS) matrix of all individuals was calculated using the command ‑‑genome on the filtered genotype data. Multidimensional scaling (MDS) analysis was performed on the IBS matrix using the eigendecomposition-based algorithm in PLINK v1.90b6.10.

After imputation, variants with a MAF <1%, an HWE test p<1×10^-6^, and an INFO metric <0.8 were removed. Imputation was conducted using SHAPEIT v2 (r837) (1), IMPUTE2 v2.3.2 (2,3), and the 1000 Genomes Phase 3 reference panel.

In total, imputed genetic data were available for 2,248 individuals.

Variants before QC: 596,861; variants after QC: 284,691; variants after imputation: 8,565,143.

# References

x

| 1. | Delaneau, O., Zagury, J.F., Machini, J. Improved whole-chromosome phasing for disease and population genetic studies. Nature Methods. 2013 Jan; 10(1): 5-6. |
| --- | --- |
| 2. | Howie, B.N., Donnelly, P., Marchini, J. A Flexible and Accurate Genotype Imputation Method for the Next Generation of Genome-Wide Association Studies. PLoS Genetics. 2009 Jun; 5(6): e1000529. |
| 3. | Howie, B., Fuchsberger, C., Stephens, M., Marchini, J., Abecasis, G.R. Fast and accurate genotype imputation in genome-wide association studies through pre-phasing. Nature Genetics. 2012 Jul; 44(8): 955-959. |
| 4. | Budde, M. et al. A Longitudinal Approach to Biological Psychiatric Research: The PsyCourse Study. American Journal of Medical Genetics Part B: Neuropsychiatric Genetics. 2018 Aug; 180(2): 89-102. |
| 5. | Meller, T. et al. Associations of schizophrenia risk genes ZNF804A and CACNA1C with schizotypy and modulation of attention in healthy subjects. Schizophrenia Research. 2019 Jun; 208: 67-75. |
| 6. | Chang, C.C. et al. Second-generation PLINK: rising to the challenge of larger and richer datasets. GigaScience. 2015 Feb; 4(1). |
| 7. | Andlauer, T.F.M. et al. Novel multiple sclerosis susceptibility loci implicated in epigenetic regulation. Science Advances. 2016 Jun; 2(6): e1501678. |

x

**Supplementary Figure 1**

| **A**  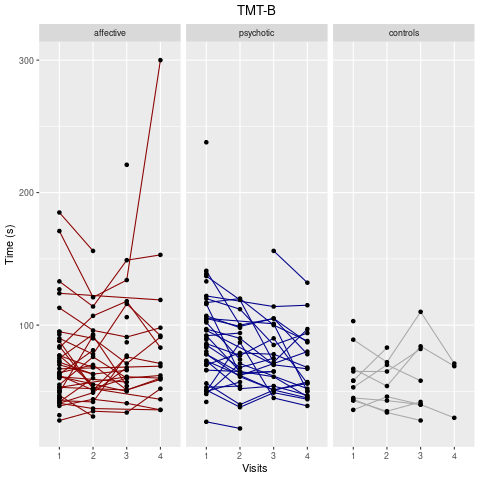 | **B**  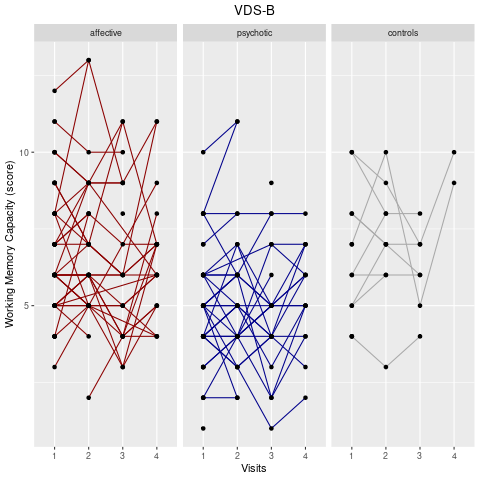 |
| --- | --- |

**Supplementary Figure 1. Spaghetti plot of the longitudinal course of approximately 320 randomly selected individuals of (A) TMT-B score (time in seconds) and (B) VDS-B score (working memory capacity) of the discovery sample. The trajectories were separated for each diagnostic group.**

**Supplementary Figure 2**

**
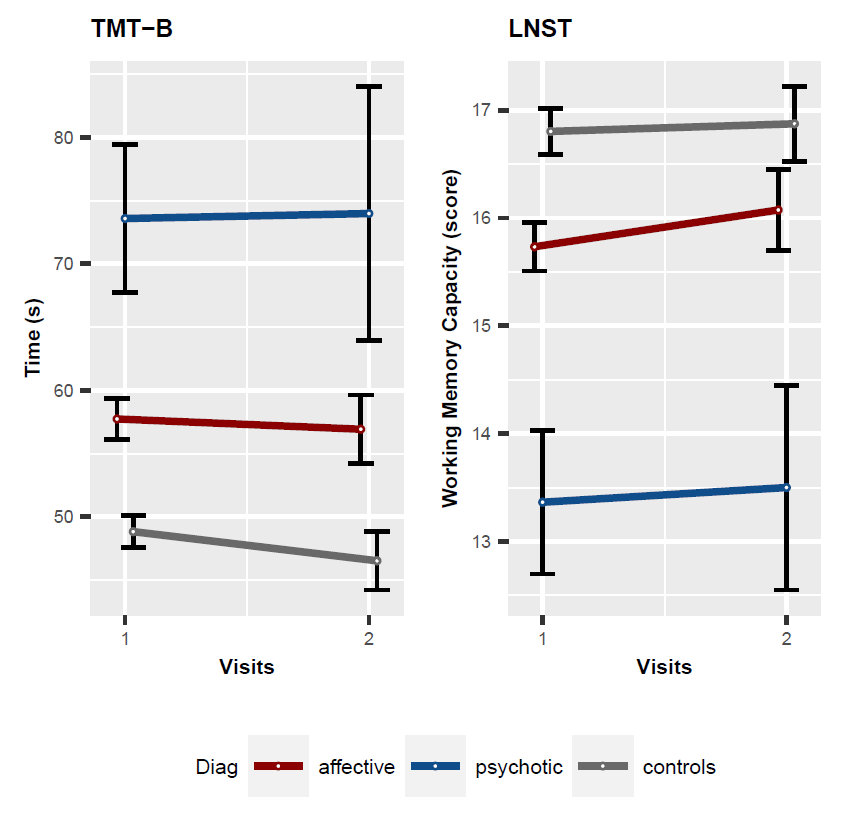
**

Supplementary Figure 2. Longitudinal course of TMT-B score (time in seconds, left) and LNST (working memory capacity, right) for each diagnostic group in the replication sample. Displayed are means with 95% confidence intervals for both visits 1 and 2, two years apart.

**Supplementary Figure 3**

| **A**  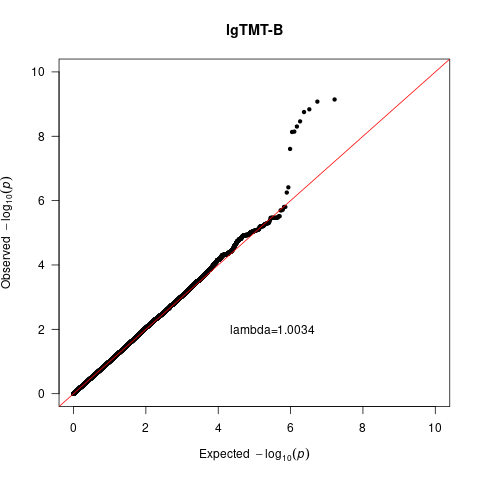 | **B**  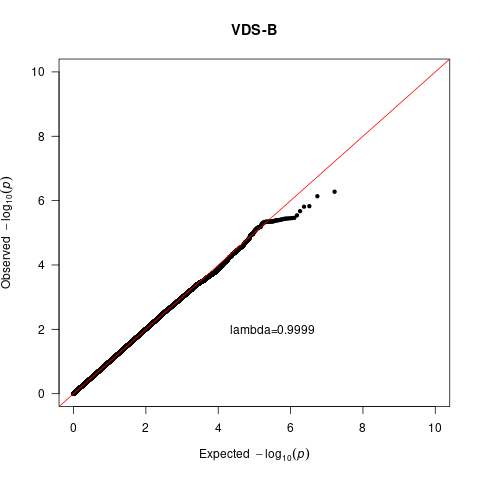 |
| --- | --- |

Supplementary Figure 3. QQ plot of the GWAS of (A) lgTMT-B and (B) VDS-B for the discovery sample with the genomic inflation factor lambda.

**Supplementary Table 1**

**Supplementary Table 1. Overview of the genome-wide significant SNPs of the GWAS of the lgTMT-B with the effect estimates** $\hat{\boldsymbol{\beta}}$ **(95% c.i.s) in original scale for SNP-by-time interaction terms. Results are given for the GWAS (LMM) in the discovery sample (DS) and for the difference analysis in the replication sample (RS). Replicated SNP (*p*_RS_ < 0.05) is in bold. The SNPs are ordered according to their location on chromosome 5, the gene context and the distance to the next gene was received with FUMA (https://fuma.ctglab.nl/).**

| **CHR** | **SNP** | **Location (BP)** | ${\hat{\boldsymbol{\beta}}}_{\boldsymbol{DS}}$  **(95% c.i.)** | **p_DS_** | ${\hat{\boldsymbol{\beta}}}_{\boldsymbol{RS}}$  **(95% c.i.)** | **p_RS_** | **MAF_DS_** | **MAF_RS_** | **A1/A2** | **Distance (BP)** | **Gene context** |
| --- | --- | --- | --- | --- | --- | --- | --- | --- | --- | --- | --- |
| 5 | rs62368988 | 63304980 | 1.15 (1.09,1.20) | 7.4x10^-09^ | 0.89 (0.79,1.01) | 0.075 | 0.016 | 0.020 | T/C | 28109 | intergenic |
| 5 | rs62369014 | 63333947 | 1.15 (1.09,1.20) | 7.2x10^-09^ | 0.89 (0.79,1.01) | 0.075 | 0.016 | 0.020 | T/G | 57076 | intergenic |
| 5 | rs62369048 | 63417124 | 1.15 (1.10,1.20) | 3.4x10^-09^ | 0.89 (0.79,1.01) | 0.074 | 0.016 | 0.020 | A/G | 44546 | intergenic |
| 5 | rs146654929 | 63470382 | 1.15 (1.10,1.20) | 5.0x10^-09^ | 0.89 (0.79,1.01) | 0.076 | 0.016 | 0.020 | G/A | 0 | RNF 180 |
| 5 | rs62372500 | 63547213 | 1.16 (1.10,1.21) | 1.5x10^-09^ | 0.89 (0.79,1.01) | 0.075 | 0.015 | 0.019 | T/C | 0 | RNF 180 |
| 5 | rs191575088 | 63606345 | 1.16 (1.10,1.21) | 8.4x10^-10^ | 0.89 (0.79,1.01) | 0.081 | 0.016 | 0.019 | G/A | 0 | RNF 180 |
| 5 | **rs150547358** | 63640195 | **1.16 (1.11,1.22)** | **7.2x10^-10^** | **0.85 (0.74,0.97)** | **0.015** | **0.015** | **0.018** | C/A | 0 | RNF 180 |
| 5 | 5:63686382:AT | 63686382 | 1.15 (1.10,1.21) | 1.8x10^-09^ | 0.90 (0.80,1.02) | 0.112 | 0.016 | 0.020 | A/AT | 13956 | intergenic |
| 5 | rs62369421 | 63722706 | 1.14 (1.08,1.19) | 2.5x10^-08^ | 0.90 (0.80,1.01) | 0.064 | 0.017 | 0.022 | T/G | 0 | ncRNA_intronic |

**Supplementary Figure 4**

| **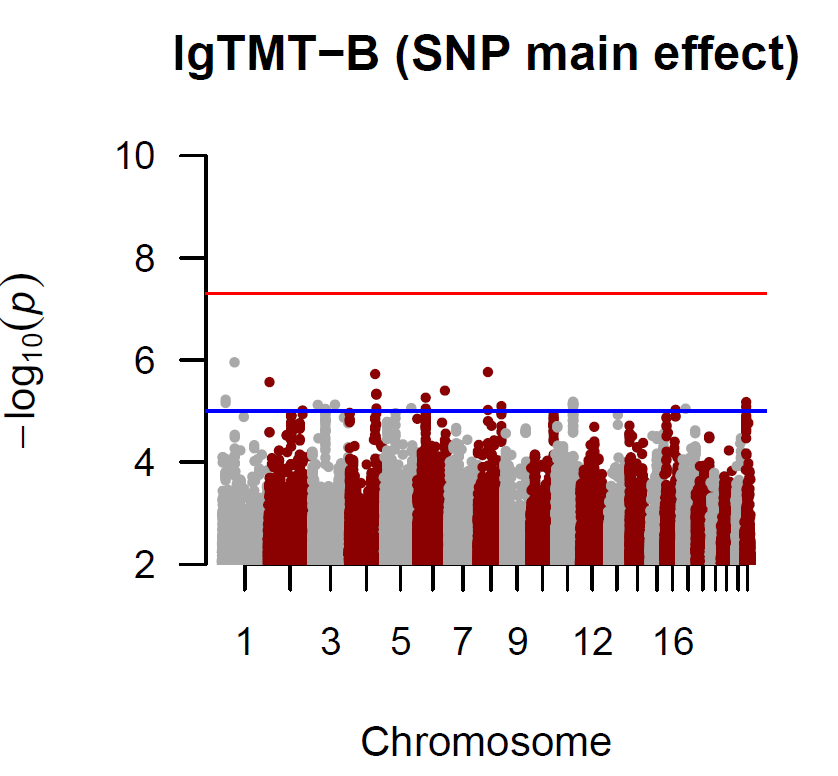** | **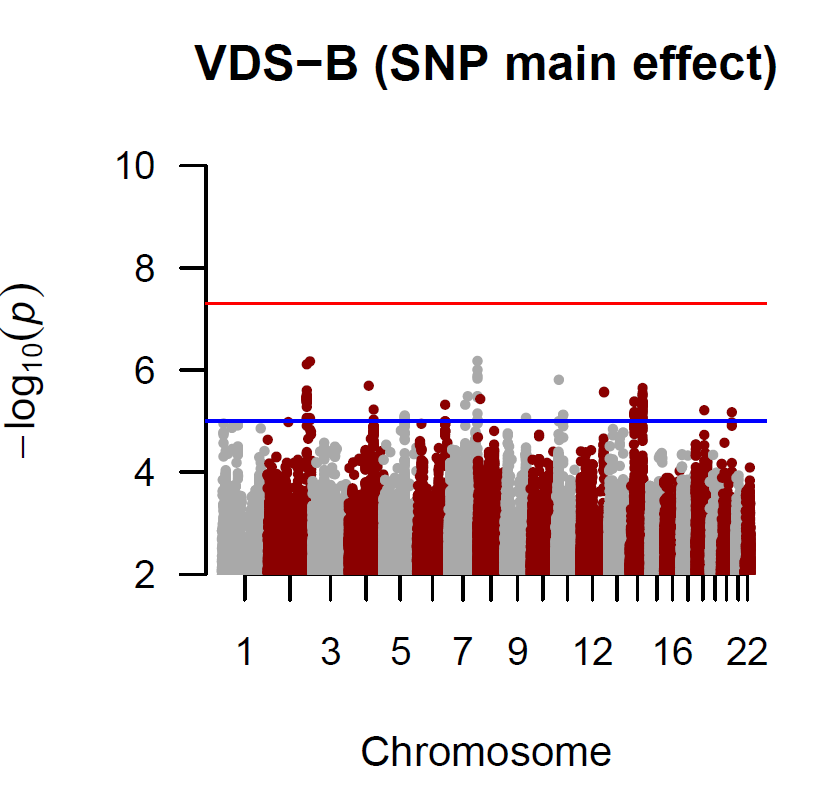** |
| --- | --- |

**Supplementary Figure 4. Manhattan plot of the GWAS of lgTMT-B (left) and the VDS-B (right) in the discovery sample testing the SNP main effect. The lines indicate the thresholds for genome-wide significance of 5×10^-8^ (red) and for suggestive SNPs (blue, p≤ 1×10^-5^).**

**Supplementary Figure 5**


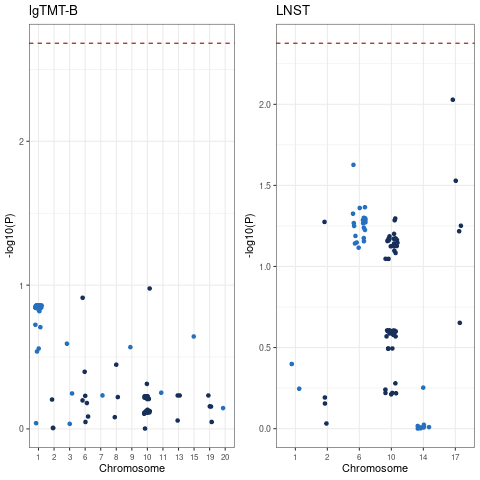


Supplementary Figure 5. Manhattan plot of the difference analysis of the SNP_NR_ (SNP not be replicated) for the lgTMT-B (left) and the LNST (right) in the replication sample (FOR2107 consortium), containing only suggestive (not significant) SNPs of the GWAS in the discovery sample (PsyCourse Study). The dashed red line presents the significance level (lgTMT-B: 0.0021; LNST: 0.0042) corrected for multiple testing.

**Supplementary Table 2**

**Supplementary Table 2. Distribution of the genotypes for SNP rs150547358 in the discovery and the replication sample. Entries refer to the number of individuals with wildtype/C-carriers in the respective diagnostic groups. The p-values marked by ^1^ are results of a Fisher’s exact test. The other p-values are results of χ^2^-tests.**

| **Visit** | **Discovery sample** | | | | | **Replication sample** | | | | |
| --- | --- | --- | --- | --- | --- | --- | --- | --- | --- | --- |
|  | **Total** | **Affective** | **Psychotic** | **Controls** | **p-value** | **Total** | **Affective** | **Psychotic** | **Controls** | **p-value** |
| 1 | 1175/34 | 486/16 | 439/13 | 250/5 | 0.4994 | 595/16 | 278/12 | 39/0 | 278/4 | 0.0984^1^ |
| 2 | 803/22 | 300/11 | 285/7 | 218/4 | 0.4429 | 595/16 | 278/12 | 39/0 | 278/4 |  |
| 3 | 638/18 | 223/8 | 242/7 | 173/3 | 0.5786^1^ |  | | | |  |
| 4 | 449/12 | 175/5 | 217/7 | 57/0 | 0.5982^1^ |  |  |  |  |  |
